# Patient self-sampling: a cornerstone of future rheumatology care?

**DOI:** 10.1007/s00296-021-04853-z

**Published:** 2021-04-10

**Authors:** Harriet Morf, Martin Krusche, Johannes Knitza

**Affiliations:** 1 Department of Internal Medicine 3-Rheumatology and Immunology, Friedrich-Alexander University Erlangen-Nürnberg, Erlangen, Germany; 2 Department of Rheumatology and Clinical Immunology, Charité - Universitätsmedizin, Berlin, Germany

**Keywords:** Self-sampling, Rheumatology, Remote care, Personalised medicine, Web survey

Dear Editor,

With great interest, we read the letter by Cleaton et al. [1] regarding the impact of social distancing on a large cohort of rheumatology patients in the UK. The authors performed a large-scale evaluation to study the impact of COVID-19 on mortality, infection rate, shielding rates and compliance. Most patients followed specialists’ advice on shielding, and the mortality rate for rheumatology patients was similar to regional reports. In another publication, the authors presented an innovative technique helping patients to self-score to stratify their own COVID-19 infection risk [2].

Confinement strategies and travel restrictions catalysed a rapid switch to remote rheumatologic consultations in many countries. Remote care in rheumatic and muscular diseases (RMDs) is a dynamic field [3], which will likely maintain importance even after the COVID-19 pandemic. Video consultations and electronic patient-reported outcomes (ePROs) have already been used in remote digital care [3]. Nevertheless, a cornerstone in diagnosis and management in routine rheumatology care is still missing.

In our opinion, self-sampling is indispensable when aiming to significantly improve patient-centred remote care in rheumatology. In rheumatology care, blood tests are crucial in establishing the correct diagnosis, and evaluating treatment safety and efficacy, and yet they represent a major reason for “non-shielding” physician visits. Self-sampling has successfully been introduced in other fields of medicine, such as diabetology [4] or anticoagulation management [5]. A pilot study showed that blood microsampling is a feasible and accurate method for monitoring hydroxychloroquine levels in rheumatoid arthritis patients [6]. Inflammatory markers, such as C-reactive protein (CRP), are especially crucial in the evaluation of inflammatory disease activity (i.e. DAS-28 or ASDAS), however, to our knowledge, only point-of-care tests [7] have yet been used.

We believe that COVID-19 will not only accelerate digitalisation in rheumatology care [3], but also in patient self-sampling. We recently reported that COVID-19 increased the acceptance of digital health applications among rheumatologists (*n* = 129) and patients (*n* = 299) in Germany [8]. Despite the current experimental status of self-sampling, we observed high acceptance rates among patients and rheumatologists. To our knowledge, this is the first published work to explore the acceptance of self-sampling in rheumatology. 128 (44%) of patients and 50 (39%) of rheumatologists were interested in using self-sampling in the future. Interestingly, male patients tended to be more in favour of self-sampling than female patients (67% compared to 43%) (*r* = 0.001, *p* = 0.001). Furthermore, patients under the age of 60 years stated that they were more likely to use self-sampling in the future (*p* = 0.05; *r* = 0.137). Especially patients with spondyloarthritis (generally younger patients) expressed a positive opinion (53.7%) concerning self-sampling. Additionally, we observed a positive correlation between the travel time to the rheumatologist and interest in self-sampling in male patients. In the case of more than 30 min travel time, we observed a significant correlation with interest in self-sampling (*p* = 0.027; *r* = 0.418).

The advancement of self-sampling in rheumatology enables need adapted, low burden, patient-centered, flexible blood collection, ultimately empowering patients, rheumatologists and researchers alike.
